# Adolescent physical activity and screen time: associations with the physical home environment

**DOI:** 10.1186/1479-5868-7-82

**Published:** 2010-11-15

**Authors:** John R Sirard, Melissa N Laska, Carrie D Patnode, Kian Farbakhsh, Leslie A Lytle

**Affiliations:** 1 Kinesiology Program, Curry School of Education, University of Virginia, Charlottesville, VA, USA; 2 Division of Epidemiology and Community Health, School of Public Health, University of Minnesota, Minneapolis, MN, USA; 3 Center for Health Research, Kaiser Permanente Northwest, 3800 N. Interstate Avenue, Portland, OR, USA

## Abstract

**Background:**

Previous research on the environment and physical activity has mostly focused on macro-scale environments, such as the neighborhood environment. There has been a paucity of research on the role of micro-scale and proximal environments, such as that of the home which may be particularly relevant for younger adolescents who have more limited independence and mobility. The purpose of this study was to describe associations between the home environment and adolescent physical activity, sedentary time, and screen time.

**Methods:**

A total of 613 parent-adolescent dyads were included in these analyses from two ongoing cohort studies. Parents completed a Physical Activity and Media Inventory (PAMI) of their home environment. Adolescent participants (49% male, 14.5 ± 1.8 years) self-reported their participation in screen time behaviors and wore an ActiGraph accelerometer for one week to assess active and sedentary time.

**Results:**

After adjusting for possible confounders, physical activity equipment density in the home was positively associated with accelerometer-measured physical activity (p < 0.01) among both males and females. Most of the PAMI-derived measures of screen media equipment in the home were positively associated with adolescent female's screen time behavior (p ≤ 0.03). In addition, the ratio of activity to media equipment was positively associated with physical activity (p = 0.04) in both males and females and negatively associated with screen time behavior for females (p < 0.01).

**Conclusions:**

The home environment was associated with physical activity and screen time behavior in adolescents and differential environmental effects for males and females were observed. Additional research is warranted to more comprehensively assess the home environment and to identify obesogenic typologies of families so that early identification of at-risk families can lead to more informed, targeted intervention efforts.

## Introduction

Ecological models are increasingly being used to understand physical activity and other health behaviors [1,2]. In particular, neighborhood environmental factors such as street networks and proximity to a variety of destinations have received much attention [3-6]. Less attention has been focused on the home environment although for many youth, especially younger adolescents who still have limited independence and mobility, the home environment provides a more proximal and relevant sphere of influence. The home environment, including areas both inside and outside (e.g., yard) that facilitate or discourage activity, may have an important influence on physical activity and sedentary time.

One might expect that the presence of opportunities to be active (such as sport or recreation equipment in a home) or sedentary (such as the number of televisions or other media equipment in a home) would impact levels of activity and sedentary time through their role in cueing behavior and providing ready access. These associations have been examined in college age students, but the relationships have been weak or non-existent [7,8]. In youth, the associations have also been weak or inconsistent, and research has often been limited by assessing youth's perceptions of access and availability of activity-related equipment and media opportunities rather than using more objective measures [9-12]. In addition, methodological issues related to poorly capturing the physical environment of one's home may be contributing to inconsistent findings.

The Physical Activity and Media Inventory (PAMI) is a tool that was developed to assess both the availability and accessibility of physical activity and media equipment in the home [13]. The PAMI underwent extensive developmental testing including examination of reliability and criterion validity. Test-retest reliability was acceptable to strong for all summary variables generated by the PAMI (physical activity equipment, ICC = 0.76 to 0.99; media equipment, ICC = 0.72 to 0.96). We evaluated criterion validity through simultaneous reporting from trained data collectors and adult family members, reports from participants and trained data collectors were strongly correlated (physical activity, 0.67 - 0.98; media, 0.79 - 0.96). To date, however, construct validity for the PAMI, showing that the factors assessed in the PAMI are related in the expected directions to health behaviors of interest, specifically physical activity, screen time, and total sedentary time, has not been established.

The purpose of this study was to examine the associations between the home physical activity and media equipment environment and physical activity, sedentary time, and screen time in adolescents. In addition, we are able to investigate the construct validity of the PAMI instrument and hypothesized that the amount and density of media equipment would be positively associated with screen time and physical activity equipment items in the home would be positively associated with levels of physical activity.

## Methods

Data for this paper are derived from baseline measurements collected for two longitudinal cohort studies designed to assess the etiology of youth obesity using an ecological model. The Identifying Determinants of Easting and Activity (IDEA) is one of several projects funded through the Transdisciplinary Research on Energetics and Cancer (TREC) initiative of the National Cancer Institute [14]. TREC-IDEA is a three-year longitudinal study to identify determinants of adolescent (11-17 years old) obesity based on the multiple levels of the social-ecological model [1,15]. The second study, Etiology of Childhood Obesity (ECHO), is also a three year longitudinal cohort study (age range 10-16 years old). Both studies have similar conceptual frameworks, use the same measurement instruments and protocols and both samples were drawn from the Minneapolis/St. Paul, Minnesota metro area. Based on these similarities, the samples were combined for these analyses.

### Subjects

The University of Minnesota's Institutional Review Board, Human Subjects Committee approved all study protocols. Participants for the TREC-IDEA study were recruited from: 1) an existing cohort of youth participating in the Minnesota Adolescent Community Cohort Tobacco Study, 2) a Minnesota Department of Motor Vehicle list restricted to the Twin Cities metro area, and 3) a convenience sample drawn from local communities using social networking, city parks and recreation bulletin boards, and fliers distributed at several school-based functions. For TREC-IDEA, a total of 349 parent/student pairs were measured at baseline (2006-2007). Additional details on recruitment procedures and study design have been reported elsewhere [14].

For ECHO, parent/child dyads were recruited from the membership of HealthPartners^®^, a large health management organization, within the seven-county metropolitan area of Minneapolis/St. Paul, Minnesota. To obtain a more diverse sample of youth, recruitment was targeted to enroll both healthy weight and overweight youth and parents and to oversample minorities. To be eligible for enrollment, youth were required to be current Health Partners members, in grades 6^th ^through 11^th ^in the fall of 2007, residing in one of the randomly selected middle or high-school districts included in the sample and have a parent willing to participate in a set of parental measures including a parent survey and the completion of the PAMI instrument.

For both studies, to obtain additional information about the home environment, youth were required to participate with one adult with whom he/she spends a significant amount of time (e.g., a parent/guardian, other relative or adult that cares for him/her on a regular basis). Due to the longitudinal design of these studies and the developmental time period covered (early to late adolescence), parent/student pairs were excluded from participating if they planned to move from the area in the next three years, had a medical condition that affects their growth (e.g., hypo/hyperthyroidism), were non-English speaking, and/or had any other physical or emotional condition that would affect their diet/activity levels or make it difficult to complete surveys or measurements. Only one parent/adolescent dyad per family was included in these analyses.

### Physical Activity and Media Inventory (PAMI)

The PAMI is a self-report inventory of the availability and accessibility of equipment and other resources that may support household members' participation in activity and sedentary behaviors [13]. Parent participants were instructed to go through each room/location in their home, and inventory the equipment present. For each room/location (including all rooms in the home, storage spaces, yard/garden, and garage), the PAMI included a list of response options, including 42 physical activity equipment items presented in alphabetical order. There also were five media equipment items listed: (1) television, (2) video cassette recorder (VCR) and/or DVD, (3) digital video recorder (DVR) and/or TiVo, (4) video game system, and (5) computer (desktop or laptop). Participants recorded the quantity of each type of item in the room/location and rated the accessibility of that item on a 4-point scale, with "put away and difficult to get to" coded as 1 and "in plain view and easy to get to" coded as a 4. A higher accessibility score indicated greater accessibility of the item. Active video games (e.g., Wii, DDR) were not included as an item on the original PAMI but during data collection several families indicated having these games through an option to write in a response. Active video games were coded as fitness equipment, similar to workout videos. The full PAMI instrument has been published previously [13].

The PAMI data were reduced to the following primary variables calculated separately for physical activity and media equipment: total number of items and the density of items in the home (total number of items divided by the total number of rooms/locations). In addition, we created variables to determine the number of televisions in the home and the number of televisions in children's bedrooms. We also created and assessed two summary scores. First, we calculated separate summary scores that accounted for availability and accessibility of the physical activity equipment (Physical activity Availability and Accessibility Summary Score (PAASS)) and media equipment (Media Availability and Accessibility Summary Score (MAASS)). A higher score reflects a greater overall presence in the home (both availability and accessibility) [13]. To provide more detail, we calculated the PAASS for specific categories of items, including; sports equipment, fitness equipment, transportation equipment, water sports equipment, and outdoor/yard equipment. To rank the overall quality of the home, an overall home environment score was also calculated as the ratio of the PAASS to the MAASS (referred to as the Activity:Media Ratio Score). A higher overall Activity:Media Ratio Score would reflect a home more conducive for being physically active and less sedentary.

### Physical Activity, Sedentary Time and Screen Time

Physical activity and sedentary time were assessed with the ActiGraph (Model 7164; Pensacola, FL) accelerometer. The ActiGraph has been previously validated for use with youth in laboratory and field settings [16-18]. It is a small (5.1 × 3.8 × 1.5 cm), lightweight (42.6 g), single plane (vertical) accelerometer that collects and stores accelerations from 0.05-2.00 G with a frequency response of 0.25-2.50 Hz. These settings capture normal human motion but will filter out high frequency vibrations from mechanical sources (e.g., operating a lawn mower). [19] The analog acceleration is filtered and converted to a digital signal and this value (count) is stored in user-specified time intervals. Thirty-second intervals were used for this study. ActiGraph monitors were initialized to begin collecting data at 5:00 am the day following the clinic visit where surveys and physical measures were completed [14]. Adolescents were instructed to wear the accelerometer on their right hip for seven days (except for sleeping, swimming or bathing) and to return the unit in a pre-paid courier service envelope following the data collection period. Upon return, each monitor was downloaded to a computer for subsequent data reduction and analysis.

ActiGraph data were reduced using a custom-developed software program [20,21]. All data contained within the time frame from when the monitor was initialized until the same time the following week was processed. For example, if students received their monitors at 9:00 AM on Friday the data from Friday at 9:00 until the next Friday at 9:00 would be processed through the program. For days 2-7, all data from 00:01 until midnight was reduced to summary variables. Day one and day eight were combined to form a composite seventh day of data.

Data files were scanned for data points ≥7,500 counts/30 seconds to identify implausible bodily movement or ActiGraph malfunction; no data points met this criterion. Daily inclusion criteria were established to determine days and times with acceptable accelerometer data. Blocks of time incorporating at least 30 continuous minutes of "0" output from the ActiGraph were considered to be times when the subject was not wearing the monitor [21]. These data points were eliminated and not used in any calculations. Following these deletions, days with less than 10 hours of data were eliminated from data reduction to account for unrepresentative days of physical activity. Lastly, students with at least four out of seven days of ActiGraph data were retained for the analysis sample (n = 613, 86%) [22].

After processing the data through the exclusion criteria, summary variables were calculated. Time spent in moderate to vigorous physical activity (MVPA) and sedentary times (SED) were calculated as the average number of minutes per day spent in MVPA and SED, respectively. Due to the wide age range of the participants, recently developed age-adjusted cutoffs (using single year increments) were used to classify accelerometer data into intensity categories [23].

Measuring sedentary time via accelerometry captures all sedentary activities including sitting at school, reading/doing homework, crafts and other developmentally appropriate activities but does not specifically identify screen time. Screen time is one very prevalent sedentary behavior, and one particularly associated with obesity [24]. In order to specifically measure screen media behaviors (or screen time), adolescents completed a self-administered survey in which they reported usual weekday and weekend time spent in the following activities: watching TV, watching DVDs or videos, Nintendo/Play Station/computer games, internet/computers. Response options ranged from "None" to "6+ hours per day" [25-28]. A weighted average of weekday and weekend screen media use was calculated to estimate mean hours per day spent using screen media. To be consistent with accelerometer-derived variables, screen time was converted to mean minutes per day.

### Demographic Information

Heights and weights of parents and their children were obtained during a 2-hour clinic visit. Measurements were made by trained staff using a direct read portable stadiometer (Shorr Productions, Olney, MD) for height and an electronic scale/body composition analyzer (Tanita TBF-200A; Tanita Corporation of America, Inc., Arlington Heights, IL) for weight. For the adolescents, age and gender specific BMI% were calculated using the 2000 CDC growth charts http://www.cdc.gov/growthcharts/percentile_data_files.htm.

Parents of adolescent participants completed a survey that included questions regarding the highest education level attained for all adults in the home. In addition, parents reported whether or not the child in the study received free or reduced price school lunch (yes/no), an indicator of family income. Parents also reported the number of adults (≥18 years old) and children in the home. Adolescents reported their age (in years) and their race/ethnicity.

### Statistical Analysis

Baseline data from the IDEA (2006-2007) and ECHO (2007-2008) studies were included in these analyses. All analyses were conducted with SAS version 9.1 (Cary, NC). Since boys are typically more active than similarly-aged girls and since the associations between the home environment and activity may be gender specific, the sample was stratified by gender and analyses were performed separately. Means (standard deviations) and percents were calculated to describe the sample and the distribution of values for the dependent and independent variables. Pearson correlation coefficients were calculated to determine bivariate associations between home environment variables (from the PAMI) and MVPA, SED, and screen time. Non-normally distributed variables were log transformed to more closely represent a normal distribution. If the distribution remained non-normal after the transformation, Spearman correlation coefficients were calculated using the non-transformed values. Home environment variables that were associated with MVPA, SED, or screen time behavior at the *P *≤ 0.05 level were included in the multivariable general estimating equation (GEE) models. Participants sampled from the same school may appear to be more similar than those from other schools. GEE regression models were used to account for these contextual effects and clustering within our sample, although the interpretation of these coefficients is the same as for standard linear regression coefficients. These models included age, race/ethnicity, highest level of parent education, FRPL status, number of people in the home, parent BMI, month of data collection (to control for seasonal effects on activity and accessibility of some equipment items), and gender, and also examined possible gender interactions with PAMI variables. The GEE models also accounted for clustering at the school level since a number of students shared similar school environments. Finally, we also included the study (TREC IDEA or ECHO) as a fixed effect to account for possible sample differences due to different recruitment methods.

## Results

Table 1 contains descriptive statistics for demographic, MVPA, SED, screen time and PAMI-derived variables for the total sample and by gender (N = 575). Approximately half of the sample was male and 83.6% of the sample was Caucasian with a mean age of 14.5 ± 1.8 years. The majority of students came from homes with at least one college-educated parent, and most homes had two parents and two children.

**Table 1:** Subject characteristics and descriptive statistics for physical activity and sedentary behavior; Mean (SD) or percent

	Total	Males	Females
Variable	(N = 575)	(N = 286)	(N = 289)
**Age**	14.5 (1.8)	14.5 (1.8)	14.5 (1.8)
**Race (% Caucasian)**	83.6	84.6	82.7
**BMI%**	61.1 (28.7)	61.2 (30.2)	60.9 (27.1)
**Overweight and obese (% ≥85%)^**	27.1	29.0	25.3
**Adult BMI**	27.1 (6.1)	27.4 (5.8)	26.9 (6.4)
**Highest Adult Education**			
% ≥College graduate	65.6	67.5	63.7
**Number of people in home**	4.3 (1.3)	4.3 (1.3)	4.4 (1.3)
**Number of adults in home (>18 yrs)**			
1 Adult	12.5	12.2	12.8
2 Adults	64.4	67.1	61.6
3 or More Adults	23.1	20.6	25.6
**Number of minors in home (<18 yrs)**			
1 child	27.6	28.3	27.0
2 children	42.8	44.1	41.5
3 or children	29.0	27.6	30.5
**PAMI-Derived Variables**			
PA Items*	73.0 (42.0)	79.4 (45.2)	66.8 (37.7)
PA Density*	5.1 (2.9)	5.5 (3.1)	4.7 (2.6)
PAASS*	240.3 (142.0)	262.2 (153.6)	218.7 (126.2)
PAASS Equipment Categories			
Sports*	158.7 (108.3)	179.1 (122.4)	138.6 (88.1)
Fitness	29.3 (25.7)	27.5 (22.0)	31.0 (28.9)
Transportation*	21.0 (13.5)	22.9 (13.9)	19.2 (12.9)
Outdoor	31.3 (29.5)	32.6 (30.7)	30.0 (28.3)
Media Items	10.6 (4.5)	10.8 (4.6)	10.4 (4.3)
Media Density	0.8 (0.3)	0.8 (0.4)	0.7 (0.3)
MAASS	40.6 (17.2)	41.7 (18.2)	39.6 (16.0)
Total TVs in Home	3.6 (1.8)	3.6 (1.9)	3.6 (1.7)
Total TVs in Children's Bedrooms	0.6 (0.9)	0.6 (0.9)	0.6 (0.8)
Activity:Media Ratio Score*	6.9 (5.8)	7.6 (6.8)	6.3 (4.3)
**Physical Activity and Sedentary Behavior**			
Ave Min MVPA* (ActiGraph)	43.6 (25.9)	49.7 (28.6)	37.5 (21.4)
Ave Min SED (ActiGraph)	562.9 (105.7)	555.7 (110.5)	570.0 (100.3)
Ave Min Screen Time* (Self-Report)	330.2 (219.8)	357.3 (223.2)	303.5 (213.4)

^ based on the CDC BMI growth charts

* significant gender difference, *P *< 0.05

PAASS: Physical Activity Availability and Accessibility Summary Score

MAASS: Media Availability and Accessibility Summary Score

Males accumulated more minutes of MVPA per day, compared to females. While total minutes of SED assessed by the accelerometer was similar between males and females (562.9 ± 105.7 minutes per day), males did report one additional hour of screen time per day, compared to females.

The number and density of physical activity equipment items, the PAASS and the activity:media ratio score were greater in homes with male versus female adolescents. There were no observed gender differences for the media equipment number, density or MAASS. The PAASS for the additional sports and transportation physical activity equipment categories were also greater for males than females.

On average, participants reported 14 ± 2.1 locations or rooms (range, 5 to 19) as part of their home environment. For physical activity equipment, 39% of the items were located in the garage, 15% in storage/attic/basement areas, 11% in hallways/entryways/porches/decks, and 8% in child bedroom(s). The remaining locations had 1% to 5% of the physical activity equipment items. Homes averaged 10.5 ± 4.5 media equipment items (0.8 ± 0.3 media items per location). Eighteen percent of the items were located in the child bedroom(s), 17% in the living room/dining room, 16% in the family room, 14% in the adult bedroom(s), and 12% in storage/attic/basement areas. The remaining locations had 0% to 8% of the media equipment items.

The unadjusted correlations between MVPA and the home environment variables were generally low but in the hypothesized direction and statistically significant (Table 2). PA density, PAASS, the activity:media ratio and sports equipment were significantly and positively correlated with MVPA for both males and females. PAASS scores for fitness and water/pool sports equipment were not associated with MVPA for males or females. Outdoor equipment was positively associated with females' MVPA (r = 0.12) but not males' MVPA. Sedentary time measured by the accelerometer was significantly negatively associated with the activity:media ratio score for males only. That is, the higher the ratio of activity-to-media equipment in the home, the less the sedentary time.

**Table 2:** Pearson/Spearman correlations between PAMI-derived variables and minutes spent in moderate-to-vigorous physical activity (MVPA), sedentary time, and screen time

	**ActiGraph MVPA Minutes**^**a**^	
Variable	Male	Female
PA Density	0.13*	0.16*
PAASS	0.15*	0.19*
PAASS for Equipment Categories		
Sports	0.17*	0.19*
Fitness	0.10	0.08
Transportation	0.01	0.11
Outdoor	0.06	0.12*
Activity:Media Ratio Score	0.12*	0.18*
	**ActiGraph Sedentary Minutes**^b^	

Media Density	-0.07	0.00
MAASS	-0.04	0.04
Activity:Media Ratio Score	-0.14*	-0.10
Total TVs in Home	-0.03	-0.01
Total TVs in Children's Bedrooms	-0.03	0.05
	**Self-Report Screen Time Minutes**^a^	

Media Density	0.09	0.20*
MAASS	0.07	0.12*
Activity:Media Ratio Score	-0.20*	-0.28*
Total TVs in Home	-0.00	0.03
Total TVs in Children's Bedrooms	0.00	0.17*

PAASS: Physical Activity Availability and Accessibility Summary Score

MAASS: Media Availability and Accessibility Summary Score

^a ^Spearman correlation coefficient

^b ^Pearson correlation coefficient

* *P *≤ 0.05

The associations between screen time and media equipment variables from the PAMI were less consistent compared to those with the physical activity equipment. Relationships between screen time (as assessed via self-reported survey items) and media-related PAMI variables showed significant associations mostly among females, with media density, the MAASS, and televisions in children's bedrooms positively associated with screen time and the ratio of activity:media equipment being negatively related to screen time.

The results of the adjusted GEE models regressing MVPA on the home environment variables are presented in Table 3. Results were collapsed across gender since no significant gender interactions were detected for MVPA. When MVPA was used as the dependent variable, total physical activity equipment density and the physical activity availability and accessibility summary score remained statistically significant after adjustment for confounding factors. The strongest association with MVPA was observed with total physical activity equipment density (β = 1.16, *P *< 0.01).

**Table 3:** Regression analyses for physical activity equipment variables predicting average minutes of moderate-to-vigorous physical activity (MVPA)

	ActiGraph MVPA Minutes		
	
Independent Variable	Coef	SE	p
PA Density	1.17	0.42	<0.01
PAASS	0.03	0.01	<0.01
PAASS for Equipment Categories			
Sports	0.04	0.01	<0.01
Transportation	0.12	0.08	0.13
Outdoor	0.04	0.03	0.18
Activity:Media Ratio Score	0.38	0.24	0.12

General estimating equation models adjusted for gender, age, race/ethnicity, highest adult education, number of people in the home, percent receiving free/reduced lunch, adult BMI, month of data collection, cohort and account for clustering at the school level.

No significant gender interactions detected.

In examining associations between the home media environment and accelerometer-determined sedentary time, the only significant association was a negative relationship between the Activity:Media Ratio Score and total sedentary time (β = -1.74, p < 0.01), as presented in Table 4.

**Table 4:** Regression analyses for PAMI media equipment variables predicting sedentary time and screen time by gender

				Self-Report Screen Time Minutes					
				
	ActiGraph Sedentary Minutes			Males			Females		
			
Independent Variable	Coef	SE	p	Coef	SE	p	Coef	SE	p
Media Density*	-3.07	11.59	0.79	3.75	30.18	0.90	112.73	38.91	<0.01
MAASS	-0.12	0.24	0.60	0.24	0.61	0.70	1.55	0.70	0.03
Activity:Media Ratio Score*	-1.74	0.60	<0.01	0.02	1.89	0.99	-7.33	2.85	0.01
TVs in Children's Bedrooms*	-6.34	5.25	0.23	-23.96	14.19	0.09	28.63	15.06	0.06

General estimating equation models adjusted for gender, age, race/ethnicity, highest adult education, number of people in the home, percent receiving free/reduced lunch, adult BMI, month of data collection, cohort and account for clustering at the school level.

MAASS: Media Availability and Accessibility Summary Score

* significant gender interactions for screen time

The results of the GEE models regressing self-reported screen time on the home environment variables are also presented in Table 4. Significant gender interactions were observed with all of the screen media equipment variables and screen time. Overall, the PAMI-derived media environment variables were not associated with screen time for males. However, media density, the media availability and accessibility score, and the activity:media ratio score were significantly associated with screen time for females; media equipment density had the strongest association (β = 112.7, SE = 38.91, *P *< 0.01).

## Discussion

The purpose of this study was to examine the associations between physical activity and media equipment availability and accessibility in the home environment and adolescent physical activity, sedentary time, and screen time. After adjusting for possible confounders, physical activity equipment density was significantly and positively associated with accelerometer-measured MVPA across all adolescents. Most of the PAMI-derived screen media equipment measures were significantly associated with screen time behavior in females, but not for males. In addition, the activity:media ratio score was significantly negatively associated with sedentary time and screen time behavior (among girls only). These findings confirm construct validity of the PAMI assessment tool and suggest that the PAMI is sensitive enough to detect important characteristics of the home environment that may impact adolescent health behavior.

To our knowledge, this is the first study to demonstrate an association between home environmental variables and physical activity behavior in adolescents. Previous research, relying on adolescents' self-reported perceptions of the availability of physical activity, fitness, or sports equipment, did not detect an association with accelerometer-measured physical activity [12] or observed associations that were counter intuitive (i.e., with perceived opportunities for sedentary time in the home being associated with greater levels of physical activity) and difficult to explain [9]. Other studies that have used a home inventory checklist have observed weak or no associations with physical activity in college students [7,8]. While checklists and the PAMI both have the limitation of being self-report instruments, a dichotomous yes/no checklist limits the amount of detail available for analysis. The findings from the current study suggest that it is not only whether an item is present or not that matters, it is how many of each item is present (i.e., density of physical activity equipment) and the accessibility of the items.

The associations between PAMI-derived physical activity equipment variables and MVPA were similar for both males and females, even though homes with a male study participant were more supportive of physical activity (greater physical activity equipment density and PAASS) compared to homes with a female study participant. We did not ask if other children in the home were males or females and it remains unclear why density of physical activity equipment varied by the gender of the participant. Even though our data showed that, on average, adolescent female participants lived in homes with a lower density of physical activity equipment, MVPA among females was still positively associated with the equipment they did have.

Other gender differences were also observed in the associations between home media equipment and screen time. While media equipment density was similar in homes of male and female adolescent participants, the home media environment was only significantly related to screen time among females, despite the fact that the males reported almost one additional hour per day of screen time. Previous research has demonstrated that youth with televisions in their bedrooms are more likely to spend more time watching television,[29] be less physically active or more sedentary and have a higher body mass index [24,30,31]. In particular, girls with a TV in their bedroom have been shown to be less likely to participate in vigorous physical activity, compared to those without a TV in their bedroom (1.8 vs. 2.5 hours/week) [29]. These previous findings and those from the current study indicate that the home media equipment environment may have potent negative behavioral and health effects, especially for girls, potentially by providing a more compelling cue to watch television. These gender differences should be examined in further observational and intervention research both in terms of looking at differential responses to environmental cues by gender but also by trying to understand why the differences exist.

This study has several strengths and limitations. Strengths include the use of accelerometers to measure time spent in active and sedentary time, and the use of the PAMI, which has demonstrated content validity and test-retest reliability [13]. Furthermore, our accelerometry data were analyzed using the most recent and widely-accepted standards for data processing. Still, the sample was predominantly Caucasian, from relatively well-educated families, and only represents one large metro area in the upper Midwest. Based on the demographic characteristics of this sample, these results may not be representative of the general population of 11-17 year old youth. Levels of MVPA in the TREC-IDEA and ECHO cohort samples were slightly greater than those measured by the National Health and Nutrition and Examination Survey conducted in 2003-2004 [32]. Compared to mean minutes of MVPA for males in the current analysis (49.9 ± 28.7), male youth from the NHANES study in a similar age group (12-15) accumulated 45.3 minutes of MVPA per day. Likewise, the mean minutes of MVPA for females in the current analysis (38.7 ± 21.9), were slightly greater than for female youth from the NHAMES study in the 12-15 age group who accumulated 24.6 minutes of MVPA per day.

This study examines only one aspect of potential factors influencing activity and screen time in youth. We did not include factors associated with the social environment (e.g. how the behaviors of other children or parents may impact behavioral choice) or individual factors (e.g. motivations to be active or preferences) in the analysis. For this research we chose to focus on one set of relationships in our ecological model, the association between the physical environment of the home and youth activity and screen time behaviors. Now that construct validity is established for this measure of the home physical environment, more complicated models including other important factors may be considered.

Also, these cross-sectional analyses cannot identify any temporal associations or causality between the home environment and behavior. It may as likely be that families with active youth have more physical activity equipment as it is that physical activity equipment cue or predispose youth to be active. Likewise, families with high media use may have more media equipment. While the magnitude of the significant associations in this study were relatively small, they were consistent and in the hypothesized direction.

## Conclusion

Our results support the hypothesis that elements of the home environment are associated with physical activity and, for girls, screen time behavior. In addition, this research demonstrates construct validity for the PAMI assessment tool in characterizing a wide array of features in the home environment. Additional research is warranted to 1) understand the differential effects of media equipment on screen time behaviors in boys and girls; 2) investigate the influence of the home physical and social environments on adolescent activity-related behaviors; 3) investigate the role of the home environment on other behaviors or physiological parameters and 4) combine the PAMI measures used here with other home environment factors to identify obesogenic typologies of families so that early identification of such families can lead to more informed, targeted intervention efforts.

## Competing interests

The authors declare that they have no competing interests.

## Authors' contributions

JRS led the development of the PAMI instrument and guided analyses and manuscript development. All co-authors (MNL, CDP, KF, and LAL) contributed to the development of the PAMI and provided critical input during data analysis and manuscript development. KF was responsible for carrying out the data analysis. LAL is the principle investigator for the TREC-IDEA and ECHO studies and, thus, was also responsible for the overall study design. All authors read and approved the final manuscript.
